# Upregulation of Phosphatidylinositol 3-Kinase (PI3K) Enhances Ethylene Biosynthesis and Accelerates Flower Senescence in Transgenic *Nicotiana tabacum* L.

**DOI:** 10.3390/ijms18071533

**Published:** 2017-07-15

**Authors:** Mohd Sabri Pak Dek, Priya Padmanabhan, Sherif Sherif, Jayasankar Subramanian, Gopinadhan Paliyath

**Affiliations:** 1 Department of Plant Agriculture, University of Guelph, Guelph, ON, N1G 2W1, Canada; mhdsabri@upm.edu.my (M.S.P.D.); padmanab@uoguelph.ca (P.P.); ssherif@vt.edu (S.S.); jsubrama@uoguelph.ca (J.S.); 2 Department of Food Science, Faculty of Food Science and Technology, Universiti Putra Malaysia, 43400 Serdang, Malaysia; 3 Alson H. Smith Jr. Agricultural Research and Extension Center, Virginia Tech, Winchester, VA 22602, USA

**Keywords:** phosphatidylinositol 3-kinase 1, tobacco flower, senescence, overexpression, ethylene signal transduction

## Abstract

Phosphatidylinositol 3-kinase (PI3K) is a key enzyme that phosphorylates phosphatidylinositol at 3’-hydroxyl position of the inositol head group initiating the generation of several phosphorylated phosphatidylinositols, collectively referred to as phosphoinositides. The function of PI3K in plant senescence and ethylene signal transduction process was studied by expression of *Solanum lycopersicum* PI3K in transgenic *Nicotiana tabacum*, and delineating its effect on flower senescence. Detached flowers of transgenic tobacco plants with overexpressed *Sl*-*PI3K* (OX) displayed accelerated senescence and reduced longevity, when compared to the flowers of wild type plants. Flowers from PI3K-overexpressing plants showed enhanced ethylene production and upregulated expression of 1-aminocyclopropane-1-carboxylic acid oxidase 1 (*ACO1*). Real time polymerase chain reaction (PCR) analysis showed that *PI3K* was expressed at a higher level in OX flowers than in the control. Seedlings of OX-lines also demonstrated a triple response phenotype with characteristic exaggerated apical hook, shorter hypocotyls and increased sensitivity to 1-aminocyclopropane-1-carboxylate than the control wild type seedlings. In floral tissue from OX-lines, *Solanum lycopersicum* phosphatidylinositol 3-kinase green fluorescent protein (PI3K-GFP) chimera protein was localized primarily in stomata, potentially in cytoplasm and membrane adjacent to stomatal pores in the guard cells. Immunoblot analysis of PI3K expression in OX lines demonstrated increased protein level compared to the control. Results of the present study suggest that PI3K plays a crucial role in senescence by enhancing ethylene biosynthesis and signaling.

## 1. Introduction

Senescence is a complex process that involves a network of molecular and enzymatic events initiated after the activation of receptors by hormones, signal transduction, gene expression, and synthesis of related proteins and enzymes, ultimately resulting in the physiological response [1]. Ethylene is the primary hormone that initiates senescence, manifested in fruit ripening and wilting of flowers. In addition, ethylene is also involved in protection from fungal pathogens, and adaptation of plants to environmental stresses [2]. Senescence in plant systems is associated with a progressive increase in ethylene biosynthesis [3,4], often showing a climacteric peak resulting from autocatalytic ethylene biosynthesis [5], as in climacteric fruits. Respiration also peaks during the increase in ethylene production. Moreover, the participation of several other key components has been proposed to be associated with senescence, including signal transduction by calcium ions and cross-talk with other growth regulators such as abscisic acid, salicylic acid, jasmonic acid and polyamines, at biochemical and genetic levels [6,7].

Ethylene signaling is initiated with the binding of ethylene to the receptors such as ETR1 [8,9,10,11], as well as with others in the receptor family including ETR4, ERS1 and EIN4 [10]. Once activated by ethylene, the receptor transfers ethylene-induced signal to downstream components through a phosphorelay mechanism [12]. A Raf-like kinase termed constitutive triple response 1 (CTR1) regulates downstream signal transduction processes from the ethylene receptor ETR1 [13]. CTR1 physically interacts with the receptor and suppresses the action of downstream components in the absence of ethylene, and this inhibition is released after binding of the receptor with ethylene. Upon binding of ethylene to its receptors such as ETR1, CTR1 has been proposed to dissociate from the receptors, thus becoming unable to continually phosphorylate EIN2 and inhibit signal transduction. When the association between receptor and CTR1 becomes terminated, the pathways downstream of CTR1 get activated, enhancing gene expression [14]. Several molecular events in downstream ethylene signaling have been unraveled; however, the immediate events that occur after ethylene perception are still under investigation.

Phosphatidylinositol 3-kinase (PI3K) is an enzyme that phosphorylates phosphatidylinositol (PI) at its 3′-hydroxyl position to produce phosphatidylinositol 3-phosphate (PI3P) [15]. Uniquely for plants, only one class (class III) of PI3K has been identified so far, as compared to the mammalian system which has two other classes. Plant PI3K has a similar protein structure domain with class III mammalian PI3K, which only phosphorylates PI into phosphatidylinositol 3-phosphate [16,17,18]. PI3K is required for normal plant growth and development [19], normal stomatal movements in response to abscisic acid [20], root hair elongation [16], and cytoskeleton arrangements [21]. In plants, PI3P plays a crucial role in membrane biogenesis and the trafficking of Golgi-derived vesicle [22], vesicle trafficking from the trans-Golgi network to the lumen of the central vacuole [23], and nuclear transcription processes [24]. *Arabidopsis* plants expressing antisense *AtVPS34* showed impaired growth and development and were unable to produce normal vacuole [19]. Mutation of *Arabidopsis thaliana* PI3K resulted in an abnormal segregation ratio in the male gametophyte and affected mitosis, also causing the formation of large vacuoles in pollen grains, suggesting the function of PI3K in vacuole reorganization and pollen development [16].

In the present study, we have demonstrated that PI3K expression can be increased during flower senescence. The coding region of *Solanum lycopersicum* PI3K was expressed in tobacco (*Nicotiana tabacum*) plants to evaluate the function of PI3K on ethylene biosynthesis and flower senescence. Morphological changes associated with the overexpression of PI3K, as well as flower senescence properties, were investigated in transgenic tobacco plants, and are presented as follows.

## 2. Results

### 2.1. Overexpression of S. lycopersicum PI3K in Nicotiana tabacum

**Figure 1:**
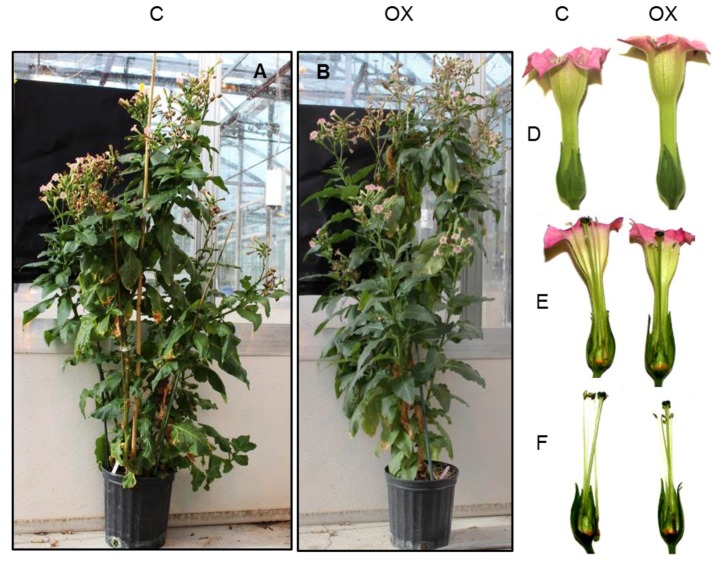
Panel **A**: Control (C), Panel **B**: transgenic tobacco (OX) plants that express *Solanum lycopersicum* phosphatidylinositol 3-kinase-Green fluorescent protein (PI3K-GFP) grown in a greenhouse. Panel **D**: Flowers from C and OX, Panel **E**: Cross section of flowers from C and OX, Panel **F**: Pistil and stamen of flowers from C and OX.

**Figure 2:**
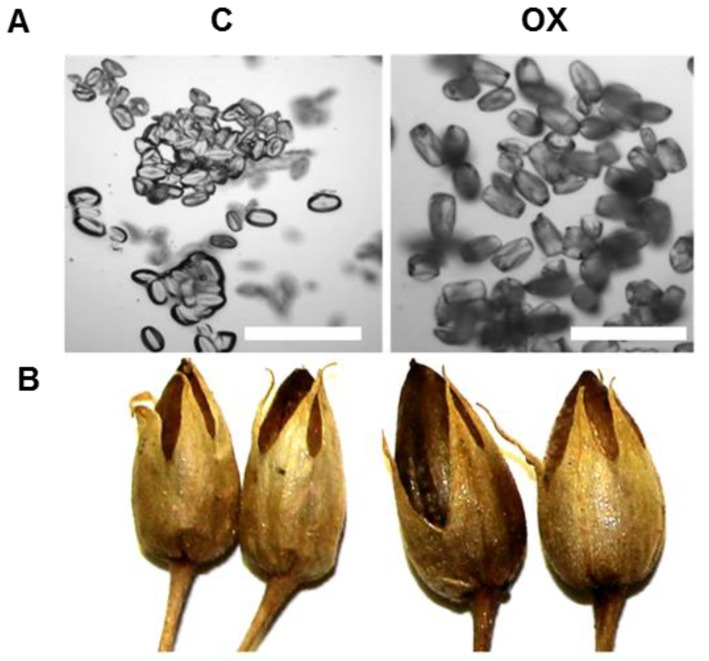
Comparison of pollen and fruit from control (C) and transgenic tobacco (OX) plants that express *Solanum lycopersicum* PI3K (PI3K-GFP). Panel **A**: Microscopic observation of pollen from control and OX flowers, Scale represent 100 µm, Panel **B**: Fruit from control and transgenic tobacco (OX) plants.

A 2448 base pair full length cDNA of PI3K designated as *Sl-PI3K* was amplified by polymerase chain reaction (PCR) from tomato cDNA, and cloned. The *Sl-PI3K* cDNA has an open reading frame of 2445 bp which encoded a polypeptide of 815 amino acids, with a predicted molecular mass of ~93 kDa. Sequence comparison of *Sl*-PI3K with *N. tabacum* PI3K (*Nt-*PI3K) showed a high degree of identity (99%) (Supplementary Figure S1 and S2). To elucidate the potential functions of PI3K in plant development, the coding sequence of *Sl-PI3K* was cloned into pGREEN vector under the control of 35S dual promoter at the C-terminus of the green fluorescent protein (GFP) coding region, for overexpression in *N. tabacum*. Twelve lines of transgenic tobacco plants were generated via *Agrobacterium*-mediated transformation. The primary putative transgenic plants were screened for transgene integration by PCR using specific primers, and three lines of transgenic tobacco plants were confirmed for transgene integration. These confirmed transgenic lines with *Sl-PI3K* (designated as PI3K-OX, overexpression lines–OX1, OX3 and OX11) were selected for further studies. Transgenic (OX) and control plants were grown in a greenhouse to compare the effects of overexpression of PI3K on general plant growth and development. A comparison of control and OX plants is shown in Figure 1 (Panels A and B). Flowers from control and transgenic lines were also compared. No distinct differences in phenotype and floral morphology were noticed among transgenic PI3K-OX plants and control under normal growth conditions (Figure 1, Panels D–F). Also, overexpression of PI3K did not adversely affect pollen development, pollen viability and seed set in transgenic tobacco plants (Figure 2). Pollen and fruit from OX plants were slightly larger than the control pollen and fruit (Figure 2).

### 2.2. PI3K Transcript Levels in Detached Tobacco Flowers and Immunoblot Analysis PI3K in Tobacco Flowers

**Figure 3:**
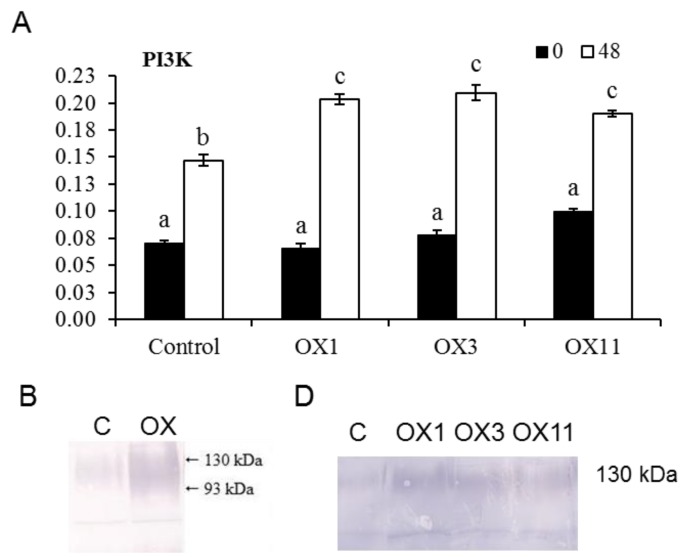
Panel **A**: Quantitative Realtime PCR analysis of transcript levels of phosphatidylinositol 3-kinase in flowers (stage 5) of control (C) and transgenic PI3K (OX1, OX3 and OX11) *N. tabacum* lines. Detached tobacco flowers were stored for 48 h for analysis. Data represents mean ± SE from three biological replicates. Analysis of variance (ANOVA) was performed on data and significant (*p* < 0.05) differences between means were determined using Duncan test. Statistical significance among means is denoted by different letters (a–c). Panel **B**: Immunoblot analysis of PI3K protein using antibodies against PI3K in control (C), and transgenic PI3K overexpressing (OX) *N. tabacum* floral tissues. The broad band represents a combination of native PI3K (~93 kDa), and PI3K-GFP (~130 kDa). Panel **D**: Immunoblot analysis performed with antibodies against GFP. Total proteins were isolated from flowers of control and transgenic PI3K (OX1, OX 3 and OX11) tobacco lines and immunoblot analysis was carried out with antibodies against GFP.

Expression of *PI3K* was also quantified in detached tobacco flowers from control and PI3K-OX lines stored for 48 h at room temperature (Figure 3A). Transcript levels of *PI3K* were similar in both control and OX flowers at the beginning (at 0 h of detachment), however *PI3K* transcript levels were found to be significantly higher in PI3K-OX lines than the control when tested after 48 h of detachment just before petal wilting symptoms were noticed.

Total protein from tobacco flower tissue was separated by SDS-PAGE and immunoblot analysis was performed with antibodies against PI3K and GFP to confirm PI3K and GFP expression in tobacco plants (Figure 3B,D). A broad band corresponding to a group of proteins ranging in molecular mass between 93 and 130 kDa was detected in high abundance in flower proteins of PI3K-OX line (Figure 3B). This band in OX plants may represent a combination of native PI3K (~93 kDa) and PI3K-GFP (~130 kDa). Protein fraction from control plants also showed expression of PI3K as revealed by a protein band with a molecular mass of ~93 kDa, though lesser in abundance than the bands corresponding to PI3K. A band of ~130 kDa corresponding to PI3K-GFP was detected in OX flowers (OX1, OX3, and OX11) with GFP antibodies which correspond to PI3K-GFP (Figure 3D). This band was not observed in control.

### 2.3. PI3K Overexpression Accelerates Tobacco Flower Senescence and Reduces Flower Lifespan

**Figure 4:**
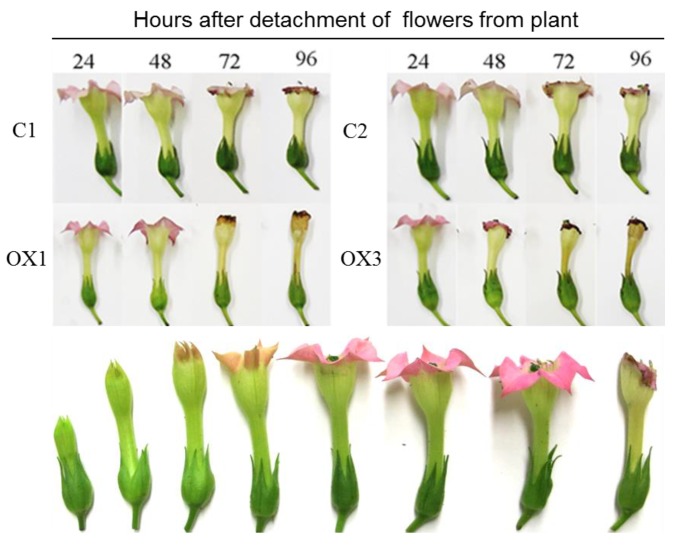
Effects of *Sl*-PI3K overexpression on senescence of detached *Nicotiana tabacum* flowers. Top panel: Detached flowers from control (C1 and C2) and transgenic tobacco lines with PI3K overexpression (OX1 and OX3) at stage 5 were stored at room temperature for 96 h in dark. Flowers were monitored and photographed every 24 h. C—Control, OX—PI3K Overexpressed. Bottom panel depicts the progressing developmental stages (1 to 8) of WT *N. tabacum* flowers.

The normal pattern of flower development and senescence of tobacco flowers (stages 1 to 8 numerically from left) is shown in the bottom panel of Figure 4. Senescence and longevity were evaluated in flowers (at stage 5) of control and transgenic PI3K-OX tobacco plants to determine the impact of PI3K overexpression (Top panel, Figure 4). As shown in Figure 4, detached flowers of OX lines senesced quickly and completely wilted after 48 h of detachment compared to control. On the contrary, control flowers remained fresh even after 48 h, but wilting started after 72 h and flowers were fully senesced by 96 h of incubation at room temperature. Therefore, flowers from PI3K-OX lines demonstrated early or accelerated senescence. Further, this accelerated flower senescence was more pronounced and noticeable in some lines, such as in OX3 rather than in OX1 (Figure 4).

### 2.4. Transgenic Tobacco Flowers Produced More Ethylene

**Figure 5:**
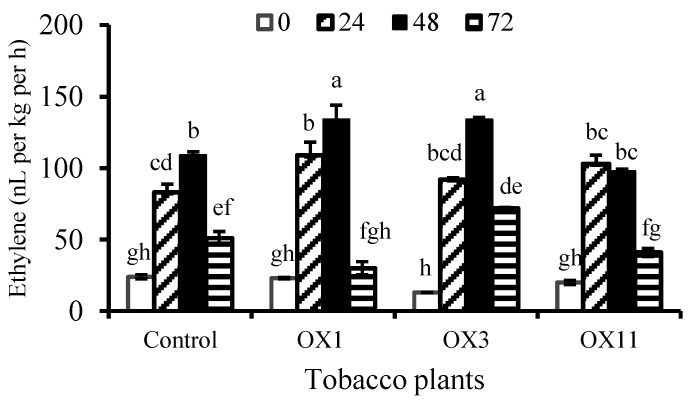
Temporal changes in ethylene production by detached *Nicotiana tabacum* flowers from control, and different PI3K-over expressed (OX) transgenic lines. Flowers selected at stage 5 were stored for 72 h after detachment from plants and the rate of ethylene production was determined every 24 h. Data shown are mean ± SE for a total of *n* = 7–10 flowers from three biological replicates. ANOVA was performed on data, and significant (*p* < 0.05) differences between means were determined using Duncan test. Statistical significance among means is denoted by different letters (a–g).

As ethylene plays a crucial role in senescence, ethylene production was also measured in flowers of PI3K-OX lines (Figure 5). In general, flowers of PI3K-OX lines released more ethylene than the control. The pattern of ethylene production was similar in both control and OX flowers. In the first 48 h of detachment of flowers of OX lines, in particular those from OX1 and OX3 released more ethylene per hour as compared to the control. Also, no significant variations were observed in the pattern of respiration of flowers between control and PI3K-OX (data not shown).

### 2.5. PI3K Overexpression Affects Transcript Levels of Ethylene Biosynthesis-Related Genes

**Figure 6:**
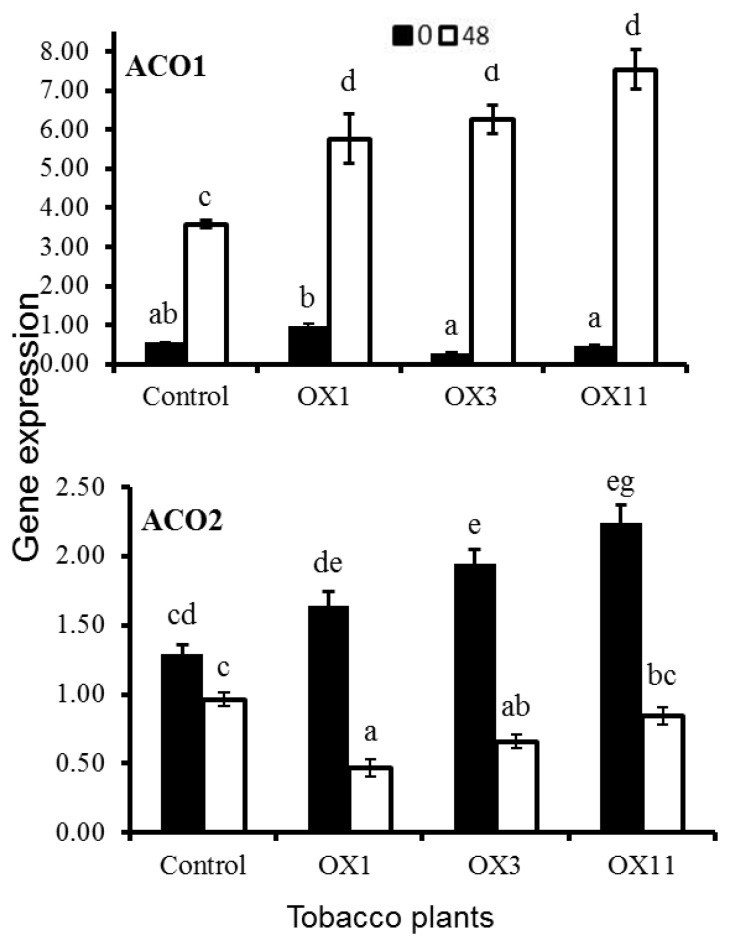
Quantitative Realtime PCR analysis of transcript levels of 1-aminocyclopropane-1-carboxylic acid oxidase 1 (*ACO1*) and *ACO2* in *Nicotiana tabacum* flowers (stage 5) of control (C) and transgenic PI3K (OX1, 3 and 11) tobacco lines. Detached flowers were stored for 48 h for analysis. Data represents mean ± SE from three biological replicates. ANOVA was performed on data and significant (*p* < 0.05) differences between means were determined using Duncan test. Statistical significance among means is denoted by different letters (a–g).

Since ethylene production in PI3K-OX flowers was higher than that of the control, transcript levels of two ethylene biosynthesis-related genes, *ACO1* and *ACO2*, were also measured in tobacco flowers 48 h after detachment from the plant (Figure 6). In ethylene sensitive floral systems, 1-aminocyclopropane-1-carboxylic acid synthase (*ACS*) and 1-aminocyclopropane-1-carboxylic acid oxidase (*ACO*) are two major genes implicated in ethylene biosynthesis. Higher expression of *Nt-ACO1* was noticed in OX lines than the control at 48 h of detachment, while the expression was almost similar in both control and OX3 plants during the initial stage (0 h). Analysis of *ACO2* revealed a higher expression in all PI3K-OX lines at 0 h than at 48 h, compared to the control. These results suggest that enhanced ethylene synthesis might be accelerating the senescence of flower.

### 2.6. PI3K-OX Tobacco Seedlings Displays Triple Response

**Figure 7:**
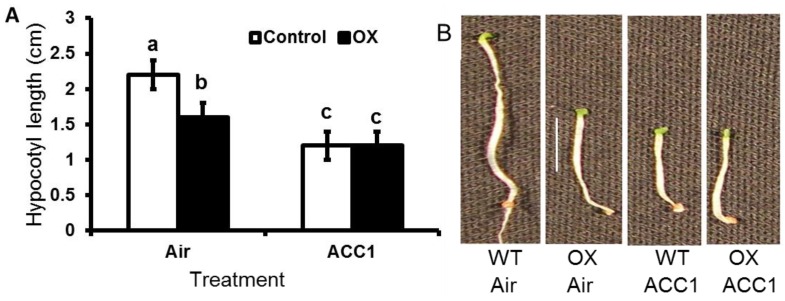
A comparison of triple response in control and PI3K overexpressing tobacco seedlings (OX). Seeds were germinated and grown for 10 days in 0 µM (air) and in 1 µM ACC (1-aminocyclopropane-1-carboxylic acid). (**A**) Hypocotyl length of tobacco seedlings after 10 days of treatment. Data represent mean ± standard error for a total of *n* = 7–10 seedlings from three biological replicates. ANOVA was performed on data and significant (*p* < 0.05) differences between means were determined using Duncan test. Statistical significance among means is denoted by different letters (a–c); (**B**) Appearance of seedlings subjected to different treatments after 10 days. Scale represent 1 cm.

Seeds of both control and PI3K-OX lines were germinated in the presence of 1-aminocyclopropane-1-carboxylic acid (ACC), which is the substrate of ACS to investigate the effect of PI3K overexpression in transgenic tobacco plants. Control seedlings showed elongated hypocotyl in the absence of ACC, while PI3K overexpressed seedling showed shortened hypocotyl and stunted root suggestive of overactive ethylene response (Figure 7). In the presence of ACC, both etiolated control and PI3K-OX seedlings showed the triple response phenotype characterized by shortened hypocotyls, stunted root and the formation of apical hook. These results suggest that PI3K action occurs downstream of the ethylene receptor.

### 2.7. Subcellular Localization of PI3K in Tobacco Seedlings

**Figure 8:**
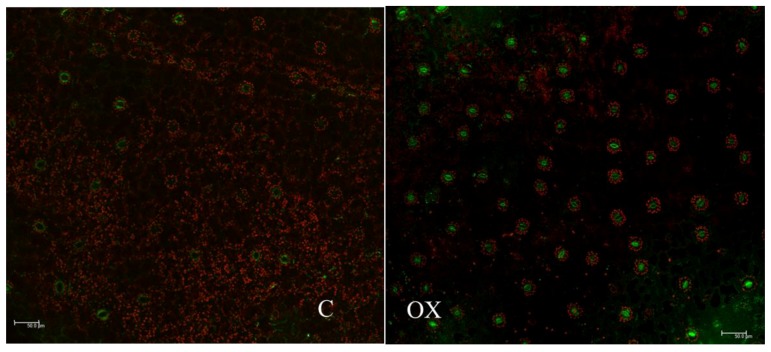
Localization of GFP in flowers of vector control (transformed with 35S-GFP construct) and PI3K-GFP overexpressed *Nicotiana tabacum* by confocal laser scanning microscopy. Control (C) and PI3K-overexpression (OX) line. GFP fluorescence is clearly visible in the guard cells of flowers from PI3K overexpressed plants. Scale represents 50.0 µm.

**Figure 9:**
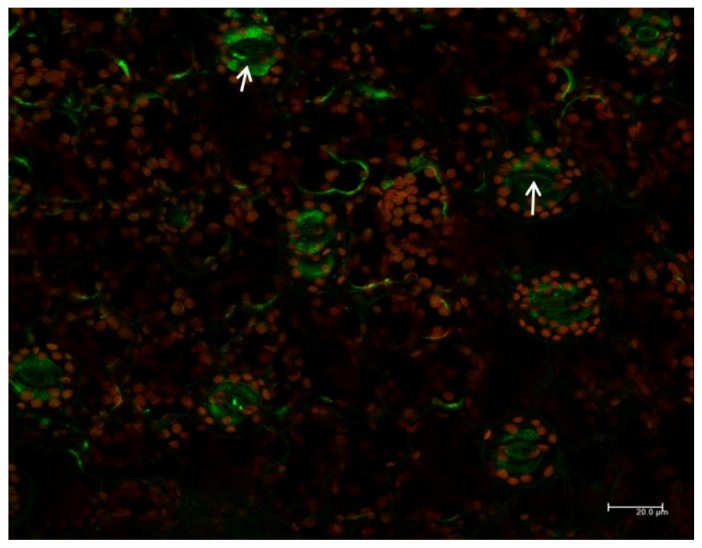
Localization of phosphatidylinositol 3-kinase (PI3K-GFP) in the cytoplasm and inner membrane of guard cells in transgenic (OX) *Nicotiana tabacum* by confocal laser scanning microscopy. Arrows denote regions showing expression in cytoplasm (Top left) and in the inner membrane of guard cell (Bottom, right). Scale represent 20.0 µm.

In floral tissues of PI3K-OX tobacco, fluorescence associated with PI3K-GFP was localized mainly in the inner membrane of guard cells. This GFP signal was very intense in the guard cells (Figure 8, Panel OX and Figure 9). PI3K-GFP was also localized in the cytoplasm, and potentially in endomembrane (Figure 9, arrow).

## 3. Discussion

### 3.1. PI3K in Flower Senescence

Phosphatidylinositol 3-kinase is a ubiquitous enzyme present in both plants and animals, indicating their functional significance in growth and developmental processes. PI3K has been identified as a key member of the signal transduction pathway in mammals, and as a major target for pharmacological interventions. Previous studies have shown that PI3K plays vital roles in many aspects of plant growth and development, including stomatal movements [20], vesicle trafficking [23], pollen development [25], and so on. The presence of only a single copy of the PI3K gene and its ubiquitous occurrence in plants points to its multiple roles in plant growth and development. *Arabidopsis* plants expressing antisense PI3K showed a lethal phenotypic response, indicating that PI3K action is critical to developmental roles in plants [19].

PI3K has been reported as a vital enzyme for plant development. PI3K has been localized in cytosol, central vacuole of stomata, and in endosomal vesicles that are close to Golgi stacks [22]. Similarly, PI3P has been found to localize in internal vesicles of endosomes, and vacuoles in yeast cells. These results suggest the role of PI3K in the early stage of endosome formation, in particular, prior to the formation of multivesicular structures [26]. The present study showed the localization of PI3K in cytoplasm and in inner membrane of guard cells (Figure 8 and Figure 9). These observations are in parallel with other reports where PI3K has been localized in multiple organs [16,20,22]. In PI3K-OX plants, strong GFP signals were detected in the guard cells particularly concentrated towards the inner membrane (Figure 9), which is similar to previous observations on the involvement of PI3K and its product PI3P in guard cell function [20,27]. Decreased stomatal closure in response to abscisic acid was detected in guard cells overexpressing a PI3P-binding protein, and also in response to treatment with PI3K inhibitors [20]. The localization of PI3K at the plasma membrane supports the function of phosphatidylinositol signaling which starts at the plasma membrane, and subsequently directs the localization of auxin efflux facilitators during early stage of root gravitropism before root curvature [28].

The mechanism of action of PI3K in plant senescence has not been previously investigated. An interesting aspect that becomes apparent from this study is that overexpression of PI3K-GFP in tobacco accelerated various parameters that are characteristic to flower senescence, suggesting its participation in ethylene signal transduction. From visible morphological symptoms, overexpression resulted in acceleration of senescence by ~24 h, which normally occurs in 72 h in the control. In addition, climacteric like ethylene production, respiration, and expression of *ACO2* were accelerated in PI3K overexpressed flowers as compared to the control. Since ethylene biosynthesis in plants is tightly regulated via positive feedback regulations upon ethylene binding to its receptors, the initial synthesis of ethylene triggers an autocatalytic cascade of events, increasing ethylene biosynthesis manifested as the climacteric [29,30]. Flowers from OX plants produced significantly higher levels of ethylene during 48 h as compared to the control. The expression of ethylene biosynthesis-related genes *ACO* and *ACS* are often correlated with flower senescence [31]. Transcript levels of *ACO1* were considerably low in both control and OX flowers at the beginning of incubation, which increased several-fold during 48 h of incubation. *ACO2* expression was relatively high at the beginning of incubation, but decreased during 48 h of incubation. Senescence in carnation flowers is temporally related to autocatalytic ethylene production and petal in-rolling [32,33]. Enhanced ethylene production during senescence of carnation flowers was associated with increased expression of *ACO* genes in senescing petals [34]. Enhanced expression of *ACO1* might be contributing towards the increased production of ethylene in PI3K-OX plants and accelerating flower senescence. These results suggest that PI3K has a key role in the signal transduction process which leads to activation of ethylene biosynthesis.

Another piece of evidence that supported the involvement of PI3K in mediating ethylene response was obtained from the spontaneous triple response exhibited by the dark grown seedlings of transgenic OX tobacco seedlings (Figure 7). Triple response is a simple phenotypic test for identifying the constitutive expression of ethylene related growth effects in plants [35]. Dark grown seedlings in general show an elongated hypocotyl, apical hook and etiolation. In the presence of ethylene, an exaggeration of the apical hook, a radially swollen short hypocotyl, and development of shorter roots can be noticed. PI3K overexpression resulted in seedlings showing triple response phenotype in the dark in the absence of ACC, an ethylene precursor, and might be caused by spontaneously elevated ethylene production by the PI3K-OX seedlings (Figure 7). The transcript level for *ACO2* was high in flowers of OX plants which may have helped in the spontaneous ethylene production that caused the triple response. This observation again suggested that ethylene production and signaling are activated in the PI3K overexpressed plants.

### 3.2. Proposed Mode of Action of PI3K in Ethylene Signal Transduction

Ethylene signal transduction involves several components that are typical to other hormones, growth regulators and environmental cues. The primary signal is received by a receptor, analogous to a bacterial two-component system which transduces the signal via response regulators and receivers and into secondary messengers such as calcium, inositol trisphosphate, and phosphatidic acid (PA). The unique feature of ethylene signal transduction is the major regulatory role of CTR1 in controlling downstream ethylene signal transduction events, which, in the absence of ethylene continuously downregulates ethylene action. The activity of CTR1 can be controlled by the phospholipase D (PLD) action in the membrane, and binding of the PA generated to the kinase domain of CTR1 causing its inactivation (Raf-like kinase) [36]. So the activity of CTR1 may be controlled through the level of PA in the membrane and thus, the ratio of active CTR1 to inactive CTR1 (bound with PA), may control the intensity of downstream signaling and release from suppression. The inhibitory effect of PA on CTR1 may directly affect the ethylene signal transduction pathway [37]. So the amount of PA might be a regulator of CTR1 activity and ethylene signaling. Therefore, increased PLD activity can result in increased PA levels in the membrane, which may regulate the CTR1 activity and downstream signalling.

Irrespective of the recognized effects of membrane catabolism on senescence, very few studies have focused on the relationships between membrane lipid catabolizing enzymes in the initiation of senescence and ethylene signal transduction processes. Studies in our lab have delineated several of these steps involving PLD in multiple plant systems, and the physical and molecular aspects associated with ethylene signal transduction [38,39,40,41,42]. PLD has been identified as the key enzyme which initiates a series of catabolic cascades that lead to the eventual deterioration of the membrane that occurs during senescence and ripening. In recent studies, we have observed that the C2 domain that directs cytosolic PLD to the membrane has a high affinity towards negatively charged lipids such as phosphoinositides and PA. Though phosphatidylcholine is the most preferred substrate of PLD, PLD never binds to unilamellar vesicles prepared from phosphatidylcholine [29]. PA is present throughout the developmental stages in tomato microsomal membranes to be effective as a regulator of fruit development and senescence. Thus, generation of negatively charged domains in the membrane by the activation of PI3K may be an initial event upstream of PLD activation, and initiation of phospholipid catabolism, as was proposed earlier [40]. Once PLD action is initiated and PA domains generated, increasing cytosolic PLD may progressively bind to the PA domains propagating membrane lipid degradation, autocatalytically [39,41]. An interesting feature of PI3K is that it is normally cytosolic and is transported to the membrane (PI enriched region) in response to an unidentified signal (after ethylene receptor activation), through activation of a C2 domain (Pak-Dek, unpublished). Though the precise sequence of action of PI3K and PLD is hypothetical at present, it is clear that both the enzymes are involved in the ethylene signal transduction pathway. The direct demonstration of these possible events needs to be accomplished in future research.

An aspect we haven’t investigated in this study is the quantification of phosphorylated phosphatidylinositols such as PIP and PIP_2_. A general method that is being used to evaluate increase in these phospholipids is the evaluation of PI turnover after incorporating ^32^P labelled phosphate to the test organisms. If there is an enhanced turnover of PI and other phosphoinositides, the ratio of labelled phosphate to the absolute phosphate level will increase. However, this estimation is extremely difficult because of the extremely low level of PIP and PIP_2_ in plant tissues such as flowers [38]. During TLC separation, the phospholipids leave a trail, and the radiolabel in this trail will be higher in proportion to the amount of phospholipid in the spots. Thus, the trailing from PI can interfere with the radiolabel that is detected in PIP, as PIP has a lower, but closer retention factor (*R*_f_) to PI. Because of these potential errors, we used HPLC-MS to quantify the phospholipids and used selected ion monitoring to quantify phosphoinositide head group levels. This method also proved to be difficult. The ^32^P labeling method has also been used by others to quantify radiolabel incorporation into phosphoinositides [43]. However, it is important to evaluate specific radiolabel incorporation levels (as a percentage of phosphate level in individual phosphoinositides) to demonstrate enhanced turnover in response to primary signals.

## 4. Materials and Methods

### 4.1. Cloning of PI3K and Vector Construction

Total RNA extracted from *S. lycopersicum* pericarp tissue (RNeasy Plant kit, Qiagen, ON, Canada) was used as template for cDNA synthesis. RNA was treated with DNase I (Invitrogen, Burlington, ON, Canada) and purified using RNeasy Kit (Qiagen, Mississauga, ON, Canada) prior to cDNA synthesis. The first-strand cDNA was synthesized from 5 μg of total RNA using the RevertAid™ Premium First Strand cDNA Synthesis Kit according to manufacturer’s instructions (Fermentas, Burlington, ON, Canada). *S. lycopersicum PI3K* was amplified from cDNA using a set of gene specific oligonucleotide primers (Forward primer: 5′-CGGATCCATGAGTGGAAACGAATTCAGG-3′ and Reverse primer: 5′-ATGGATCCCACGCCAGTACTGAGC-3′) designed according to *PI3K* sequence (Solyc04g015350.2.1) in Sol Genomics [44]. Polymerase chain reaction was carried out using 1 μL of first strand cDNA ,1 X reaction buffer, 1.5 mM MgCI_2_, 0.2 μM primers and 0.5 μL Platinum Taq DNA polymerase (Invitrogen, Burlington, ON, Canada) in a total volume of 50 μL. The amplified 2445 bp *PI3K* gene fragment was cloned into a pGEM-T Easy vector (Promega, Nepean, ON, Canada) according to the manufacturer’s instructions. Plasmid containing the cloned *PI3K* was sequenced to confirm the orientation of cloning. Coding sequence of *Sl*-*PI3K* cDNA was cloned into Bam HI site of pGREEN vector at the C-terminus of the GFP coding region in between CaMV 35S dual promoter and GFP. The construct was transformed into *E. coli* (DH5-α) using heat-shock technique and positive colonies were selected by colony PCR. The alignment of insert in the constructs was further verified by sequencing of plasmid. The construct was introduced into *Agrobacterium tumefaciens* C58 by electroporation technique and positive colonies were selected by PCR.

### 4.2. Agrobacterium tumefaciens-Mediated Stable Transformation of Tobacco

*Agrobacterium tumefaciens*-mediated tobacco (*Nicotiana tabacum* L. cv. PetH4) plant transformation was carried out by leaf-disc method [45]. Tobacco plants were also transformed with pGREEN as control. Transgenic plants were selected on Murashige and Skoog [46] medium (PhytoTechnology Laboratories, Shawnee Mission, KS, USA) containing 100 μg·mL^−1^ kanamycin (Sigma-Aldrich, St Louis, MO, USA) and 300 μg·ml^−1^ timentin (PhytoTechnology Laboratories, Shawnee Mission, KS, USA). The regenerated plants were transferred to a greenhouse (25 °C, 16 h photoperiod and 90% humidity) for their further growth and development. Putative transgenic plants were screened by PCR for the presence of transgenes using genomic DNA (QIAGEN Dneasy Plant Minikit) as template and with specific primers (forward primer covered 35S dual promoter, and the reverse primer consisted of a region of green fluorescent protein at the C-terminal of the multiple cloning site of pGreen vector) according to manufacturer’s protocol (Invitrogen, Burlington, ON, Canada). Transgenic plants were grown and the phenotype and floral characteristics were assessed. Flowers and seeds were also collected from plants for further studies. To study the effect of PI3K overexpression in tobacco flower senescence, flowers at anthesis were harvested from both control and PI3K-OX plants and the pedicel of detached flowers were immediately dipped in sterile distilled water containing kanamycin (100 μg·mL^−1^). Flowers were then stored at room temperature in dark and the senescence characteristics and flower longevity were recorded at 24 h intervals for five days. Flower samples were also stored frozen at −80 °C for gene expression studies and immunoblot analysis.

### 4.3. Gene Expression Analyses

Total RNA was extracted from tobacco floral tissues and used for quantitative real time-PCR according to standard methods (Bio-Rad, Mississauga, ON, Canada). Briefly, 25 μL of reaction mixture containing 1 X iQ™ SYBR Green Supermix (Bio-Rad, Mississauga, ON), 10 ng of cDNA and 0.2 μM of specific primers to detect *PI3K*, (Forward: 3′-GTCTCCTCTTGCCCCTG AT-5′ and reverse: 5′-TCGAAATGCCAACCGTAAT-3′), Aminocyclopropane-1-carboxylate oxidase 1 *(ACO1*, forward: 5′-TTGTGAGAACTGGGGCTTC-3′ and reverse: 5′-TCCCTTTGTCATTTTCTCCA-3′), and *ACO2* (forward: 5′-GGGTGATTGCTCAGCCTC-3′ and reverse: 5′-CAACAATTGTGGTGCTGGA-3′) gene expression in *N. tabacum* plants. Amplification and quantitation was conducted using the CFX Detection System (Bio-Rad, Mississauga, ON, Canada). Cycling conditions were as follows: 94 °C for 3 min, followed by 40 cycles of 94 °C for 30 s, and 1 min at 60 °C. PCR products were analyzed using a dissociation curve from 65 °C to 95 °C. The threshold cycle (CT) values for each gene were normalized to those of *Nt*-Actin. Gene expression was calculated as 2^−Δ*C*^_T_. The measurement was carried out for three biological replicates and with three technical replicates for each sample.

### 4.4. Immunoblot Analysis

Total protein from tobacco flowers was extracted by grinding the floral tissue in extraction buffer (25 mM MES, 10 mM EDTA, 5 mM EGTA, 10 mM citric acid, 50 mM KCl and 10 mM ascorbic acid, pH 6.0, and also containing 2 mM DTT and proteinase inhibitor cocktail tablets (Thermo Scientific, Burlington, Canada) followed by collection of the supernatant after centrifugation (8000× *g*, 10 min, 4 °C). Protein concentration in the supernatant solution was determined by Bradford’s method [47] using bovine serum albumin as the standard. Total protein (~75 µg) denatured in SDS-sample loading buffer (90 °C for 2 min) were separated on 9% SDS-PAGE gel. Separated proteins were electroblotted (20 V, 16 h) onto a nitrocellulose membrane using a Mini Trans-Blot cell according to manufacturer’s instructions (Bio-Rad, Mississauga, ON, Canada) in a transfer buffer (25 mM Tris, 192 mM glycine, 0.1% (*w*/*v*) SDS and 20% *v*/*v* methanol, pH 8.3). After protein transfer, blots were incubated in blocking buffer (Tris buffered saline, TBS + 5% nonfat dry milk) for 2 h at room temperature and the blocked membranes were washed twice (10 min × 2) in TBS + 0.1% tween 20 (TBST). Washed blots were incubated in primary antibody to PI3K (PI3K mouse polyclonal to human PI3 kinase protein corresponding to amino acids 1–887, class III) and to GFP (mouse monoclonal to GFP from jellyfish *Aequorea victoria*) diluted in blocking buffer (1:1000) for 2 h followed by 5 rinses (5 min each) of blots with TBST. Antibodies were purchased from Abcam Inc., Cambridge, MA, USA. Blots were incubated in alkaline phosphatase conjugated secondary antibody (goat anti-mouse IgG H and L, AP) diluted (1:500) in TBS buffer for 2.5 h followed by 3 rinses in TBST (5 min × 3) and the signals were detected using Alkaline phosphatase conjugate substrate kit according to manufacturer’s instructions (Bio-Rad, Mississauga, ON, Canada). Molecular masses of the polypeptides were estimated from a plot of the log of molecular mass of protein standard versus migration distance. Immunoblot analysis of at least three independent blots was performed for each antibody.

### 4.5. Ethylene Triple Response Assay

Triple response assay was carried out on transgenic tobacco seeds [35]. Seeds were surface sterilized using 20% bleach for 10 min, followed by five rinses (5 min each) in sterile water. Surface-sterilized seeds were germinated on MS medium (pH 5.8 and solidified with 8.0% agar) containing 100 μg·mL^−1^ kanamycin, 300 μg·mL^−1^ timentin and supplemented with 1-aminocyclopropane-1-carboxylic acid (ACC at 0, and 1.0 μM). Seeds were left in dark for 10 days at 25 °C for germination. Upon germination, seedling characteristics such as hypocotyl length and root length were assessed and then photographed.

### 4.6. Measurement of Ethylene and Carbon Dioxide Production

Flowers at various developmental stages were collected from transgenic tobacco plants for measurement of ethylene and respiration. Briefly, flowers were weighed and kept in a 300 mL bottle, sealed for 1 h at room temperature. The head space samples (3 mL) were analyzed for ethylene and carbon dioxide using a Varian CP-3380 gas chromatograph (Varian Inc., Mississauga, ON, USA) equipped with a 0.5 mL sample loop. The samples were separated on a 15 mm × 0.32 mm Restek Rt-SPLOTTM capillary column (Chromatographic Specialties Inc., Brockville, ON, Canada) and measured with flame ionization detector. Ethylene was quantified based on peak area by comparing with commercial Standard (50 μL L^−1^). Measurements were performed at 24 h intervals.

### 4.7. Confocal Microscopy

Subcellular localization of GFP in tobacco plant tissues was carried out using a Leica TCS SP5 confocal laser scanning microscope equipped with 63× water immersion objective (Leica Microsystems, Berlin, Germany). Flowers of transgenic OX-lines and vector control were observed under microscope to localize GFP fluorescence (GFP fluorescence (excitation: 400 nm; emission: 530 nm) and chlorophyll auto fluorescence (excitation: 400 nm; emission: 650 nm)). Captured images were analyzed using the Leica Application Suite for Advanced Fluorescence.

### 4.8. Statistical Analyses

Data obtained in the study was analyzed using SPSS Version 16. Analysis of variance was performed by analysis of variance (ANOVA) and significant (*p* < 0.05) differences between means were determined using Duncan tests.

## 5. Conclusions

In conclusion, PI3K overexpression in tobacco plants resulted in accelerated flower senescence and enhanced ethylene production by transgenic flowers, compared to the wild type. Overexpression of PI3K also amplifies the triple response phenotype as compared with control seedlings, suggesting enhancement of ethylene biosynthesis and action. The present study provides the first evidence on the involvement of PI3K in plant senescence and that it may play an early event in the ethylene signal transduction process.

## Supplementary Materials

Supplementary materials can be found at www.mdpi.com/1422-0067/18/7/1533/s1.

## Abbreviations

ACO	1-aminocyclopropane-1-carboxylic acid oxidase
ACC	1-aminocyclopropane-1-carboxylic acid
PA	Phosphatidic acid
PI	Phosphatidylinositol
PI3K	Phosphatidylinositol 3-kinase
OX	Overexpression
PI3-P	Phosphatidylinositol 3-phosphate
PLD	Phospholipase D
WT	Wild type
