# Supplementary material for: Upregulation of Phosphatidylinositol 3-Kinase (PI3K) Enhances Ethylene Biosynthesis and Accelerates Flower Senescence in Transgenic Nicotiana tabacum L
Source: Int J Mol Sci. 2017 Jul 15;18(7):1533. doi: 10.3390/ijms18071533 (PMC5536021; doi:10.3390/ijms18071533)
Supplement: Supplementary file 1 [file ijms-18-01533-s001.pdf]

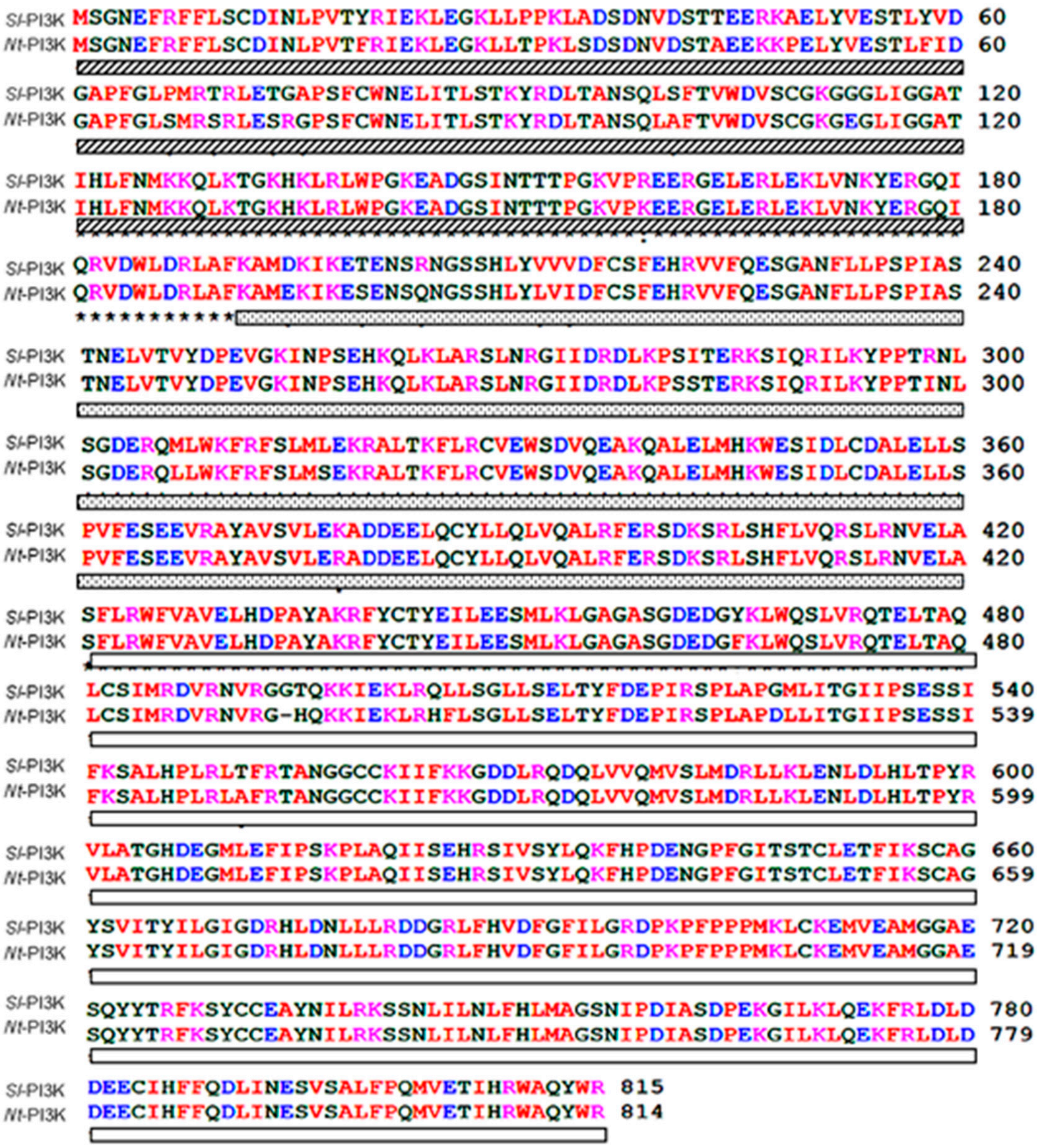

Figure S1. Amino acid alignment between *Solanum lycopersicum* PI3K and *Nicotiana tabacum* PI3K using ClustalW.

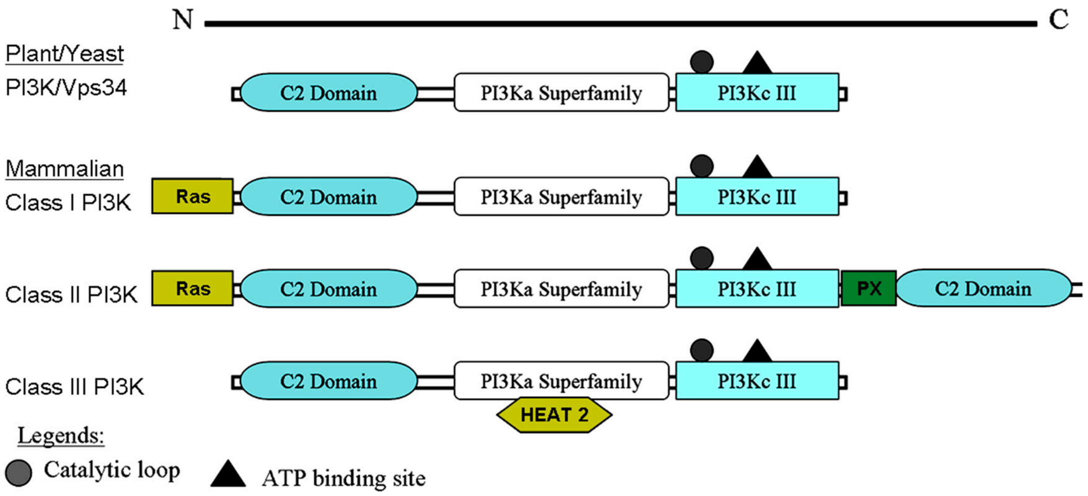

Figure S2. Structural analysis of different phosphatidylinositol 3-kinase (PI3K). The domain structures were predicted using NCBI Conserved Domain Database. Ras, ras-binding domain;PX, Phox homology domain; PI3Ka; phosphoinositide 3-kinase family, accessory domain; PI3Kc, catalytic domain of the protein kinase superfamily; HEAT, HEAT repeats.
